# Proteomic Alterations in Follicular Fluid of Human Small Antral Follicles Collected from Polycystic Ovaries—A Pilot Study

**DOI:** 10.3390/life12030391

**Published:** 2022-03-08

**Authors:** Indira Pla, Aniel Sanchez, Susanne Elisabeth Pors, Stine Gry Kristensen, Roger Appelqvist, K. Barbara Sahlin, György Marko-Varga, Claus Yding Andersen, Johan Malm

**Affiliations:** 1 Section for Clinical Chemistry, Department of Translational Medicine, Skåne University Hospital Malmö, Lund University, 205 02 Malmö, Sweden; aniel.sanchez@med.lu.se (A.S.); barbara.sahlin@med.lu.se (K.B.S.); johan.malm@med.lu.se (J.M.); 2 Clinical Protein Science & Imaging, Biomedical Centre, Department of Biomedical Engineering, Lund University, BMC D13, 221 84 Lund, Sweden; roger.appelqvist@bme.lth.se (R.A.); gyorgy.marko-varga@bme.lth.se (G.M.-V.); 3 Laboratory of Reproductive Biology, Rigshospitalet, University Hospital of Copenhagen, 2100 Copenhagen, Denmark; susanne.elisabeth.pors@regionh.dk (S.E.P.); stine.gry.kristensen@regionh.dk (S.G.K.); claus.yding.andersen@regionh.dk (C.Y.A.); 4 First Department of Surgery, Tokyo Medical University, 6-7-1 Nishishinjiku, Shinjiku-ku, Tokyo 160-8402, Japan; 5 Faculty of Health and Medical Sciences, University of Copenhagen, 1165 Copenhagen, Denmark

**Keywords:** follicular fluid, small antral follicles, proteomics, PCOS, PCO

## Abstract

Polycystic ovaries (PCO) contain antral follicles that arrest growing around 3–11 mm in diameter, perturbing the dominant follicle’s selection and the subsequent ovulatory process. Proteomic alterations of PCO follicular fluid (FF) (i.e., microenvironment in which the oocyte develops until ovulation) have been studied from large follicles in connection with oocyte pickup during ovarian stimulation. The present study aimed to detect proteomic alterations in FF from unstimulated human small antral follicles (hSAF) obtained from PCO. After performing deep-sequencing label-free proteomics on 10 PCO and 10 non-PCO FF samples from unstimulated hSAF (4.6–9.8 mm), 1436 proteins were identified, of which 115 were dysregulated in PCO FF samples. Pathways and processes related to the immune system, inflammation, and oxidative stress appeared to be upregulated in PCO, while extracellular matrix receptors interactions, the collagens-containing extracellular matrix, and the regulation of signaling were downregulated. The secreted proteins SFRP1, THBS4, and C1QC significantly decreased their expression in PCO FF, and this downregulation was suggested to affect future oocyte competence. In conclusion, our study revealed, for the first time, evidence of proteomic alterations occurring in the FF of PCO hSAF that may be related to the dysfunction of follicular growth and subsequent oocyte competence.

## 1. Introduction

Polycystic ovary syndrome (PCOS) is one of the most common causes of female infertility, with a prevalence rate between 5 and 10% [1]. A polycystic ovary (PCO) is characterized by an increased number of small antral follicles (SAF) compared with normal ovaries. Folliculogenesis is impaired, and follicles arrest prematurely when antral follicles reach a diameter of around 3–11 mm [2]. This phenomenon has been associated with the augmented production of androgen by theca cells, which is believed to be provoked by hormonal dysregulation, which includes an increase in gonadotropin-releasing hormone (GnRH) pulses that favor the upregulation of LH [3]. Although many studies have been carried out to understand the pathophysiology and molecular aspects of this disorder, specific causes of aberrant follicular development and the cessation of dominant follicle selection remain unclear.

The follicular fluid (FF) produced in antral follicles has been the subject of several studies. FF constitutes the follicular microenvironment, in which the oocyte develops until ovulation, and it is composed of secretions from the oocyte, somatic cells of the follicle (i.e., granulosa cells), and plasma proteins. The basal membrane surrounding the follicle acts as a molecular sieve that preferentially allows the passage of low molecular weight substances, while higher molecular weight substances are increasingly prevented from entering the follicular compartment [4,5,6]. In general, FF serves as a vehicle for paracrine signaling between follicular cells during folliculogenesis. Therefore, FF may act as a mirror of what happens at the molecular level in the ovary and plasma due to pathological disorders. Following this theory, proteomics has been presented as an efficient functional approach to investigate the pathophysiology of reproductive disorders like PCOS. Several proteomic studies have been conducted on ovarian FF to identify differentially expressed protein profiles in women with PCOS [7,8,9,10]. Ambekar et al. [9] were one of the first in 2015 who exploited the advances in quantitative proteomics to identify a large number of proteins in FF samples from PCOS women (770 proteins). In this study, a dysregulation of proteins involved in biological processes linked to follicular growth, oocyte competence, and ovarian steroidogenesis was found in PCOS women. These findings prompted a proposal on a mechanism for arrest in follicular growth. Another recent proteomic study focused on the protein profile of overweight/obese women with PCOS [8]. The authors detected a panel of altered proteins associated with inflammatory, immunological, and metabolic processes that could be aggravated by obesity. Specifically, the inflammatory process was deeply accessed by Li H. et al. [7], who studied exosomes derived from human FF and their association with the occurrence of PCOS.

Although relevant findings have been discovered in previous proteomic studies with PCOS samples, conclusions derived from them are tailored to the FF of preovulatory follicles collected after hormonal stimulation in connection with assisted reproductive techniques. Some of these studies have described this fact as a limitation, since one may say that the protein composition found in the samples may differ from the natural cycle [11].

To our knowledge, no large-scale proteomic studies have been performed on the FF of human SAF (hSAF) from women with PCO. The first proteomic study performed on the FF of hSAF in their natural stage was carried out by our group [12]. We demonstrated that profound and significant differences exist in FF from follicles already at a non-selected stage, predicting the ability of the enclosed oocyte to sustain meiotic resumption. A panel of proteins was linked to oocyte maturation, and a few others were cataloged as proteins possibly more concentrated or accessible in FF from hSAF compared to large follicles. In the present study, we aimed to identify proteomic alterations in the FF of unstimulated hSAF from polycystic ovaries. Consequently, we performed a label-free proteomic study using SAF fluids from unstimulated ovaries of women with and without polycystic ovaries undergoing fertility preservation.

## 2. Materials and Methods

### 2.1. Subjects

This study included ten women between 17 and 33 years of age (mean = 26.2, SD = 5.1) undergoing ovarian tissue cryopreservation for fertility preservation at the University Hospital of Copenhagen. Surplus ovarian tissue, including FFs from SAF, was donated for research by patients who gave written consent after orally conveyed information (Ethical approval: H-2-2011-044; Capital Region, Denmark). One ovary was removed laparoscopically from each woman, and the ovarian cortical tissue was isolated and cryopreserved. From the medulla tissue of each woman, two FF samples were extracted from SAF (mean = 6.9 mm, SD = 1.1) for a total of 20 FF samples. The diagnoses of the patients included three breast cancer, two sarcomas, one Hodgkin’s lymphoma, one cervical cancer, one neurological cancer, one anal cancer, and other diseases nonrelated to the ovary.

**Figure 1:**
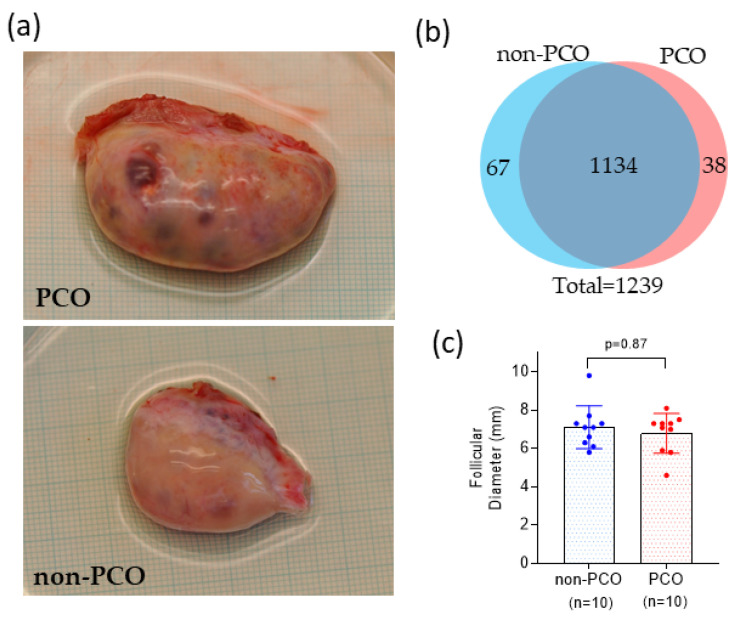
Ovarian appearance, protein quantification, and follicular size. (**a**) Examples of the polycystic (PCO) and non-polycystic (non-PCO) ovaries included in this study. (**b**) Number of proteins quantified in follicular fluid samples from small antral follicles of PCO and non-PCO ovaries. (**c**) Follicular sizes of follicles collected from PCO (mean = 6.8, SD = 1.0) and non-PCO (mean = 7.1, SD = 1.1).

Five of the ten women were characterized as having polycystic ovary (PCO), since (1) their ovaries exceeded a volume of 10 mL, (2) a polycystic appearance was observed with a high number of SAF visible on the surface of the ovary, and (3) there was an aspiration of seven or more FF. However, the number of aspirated follicles cannot be directly compared with a clinical antral follicle count (AFC) by ultrasound, as only visible and accessible antral follicles larger than 3 mm were aspirated. A clinical AFC was not available for the patients included in this study. The morphological findings of PCO were supported by hormone profiles, including increased serum AMH, LH, and an LH/FSH ratio (Table 1). On the other hand, the remaining five women were characterized as having a non-polycystic ovary (non-PCO) when a normal macroscopic appearance of the ovaries was verified (Figure 1a). A clinical diagnosis of PCOS was not conducted regularly, as the women were enrolled for fertility preservation.

### 2.2. Follicular Fluid Sample Acquisition from Small Antral Follicles

A total of 20 FF samples were collected from ten women with (*n* = 5) and without (*n* = 5) PCO. Small antral follicles (4.6–9.8 mm, mean = 6.9) exposed on the surface of the ovary or visible during the isolation of the ovarian cortex were aspirated with a 1-mL syringe fitted with a 26-gauge needle (Becton Dickinson, Brøndby, Denmark). From each ovary, two SAF were collected, resulting in two FF samples per subject. Aspiration of the FF did not affect the fertility preservation procedure. The FF samples had no visible blood contamination but were immediately centrifuged at 300× *g* for 2 min to remove debris and cells. Then, FF samples were snap-frozen in liquid nitrogen and stored at −80 °C within 30 min of sample collection. The follicular diameter was calculated based on the total volume of fluid drawn from the follicle using the calculation of the spherical shape (V = 4/3 × π × r^3^).

### 2.3. Proteomics Analysis

#### 2.3.1. Reagents and Solutions

Common chemical reagents were purchased from Sigma Aldrich (St. Louis, MO, USA). Trypsin was obtained from Promega (Madison, WI, USA). The water for the solutions was from a Milli-Q ultrapure water system (Millipore, Billerica, MA, USA). Liquid chromatography-mass spectrometry (LC-MS) grade water and organic solvents were supplied by Merck (Darmstadt, Germany).

#### 2.3.2. Sample Preparation

The quantitation of total proteins from FF was performed using the bicinchoninic acid (BCA) assay [13]. FF samples were dissolved in 1.6% sodium deoxycholate (SDC) in 50-mM NH_4_HCO_3_. The disulfide bonds were reduced and alkylated by adding dithiothreitol (DTT) to a final concentration of 10 mM (37 °C for 1 h) and iodoacetamide (25 mM, 30 min in the dark at room temperature), respectively. Tryptic digestion was performed after diluting the SDC to 0.5% at an enzyme-to-substrate ratio of 1:100 (*w*/*w*) for 16 h at 37 °C. To precipitate the SDC, 20% formic acid was added to a polypropylene filter plate with a hydrophilic polyvinylidene difluoride (PVDF) membrane (mean pore size 0.45 μm, Porvair Filtration Group, Fareham, UK) [12].

#### 2.3.3. Mass Spectrometry Data Acquisition

An UltiMate 3000 RSLCnano system coupled with the high-resolution Q Exactive HF-X mass spectrometer (Thermo Fisher Scientific, San José, CA, USA) was used. The full MS scans were set with an acquisition range of *m/z* 375–1500, resolution of 120,000 (at *m*/*z* 200), target AGC value as 3 × 106, maximum injection time of 100 ms, and normalized collision energy of 28. The top 20 precursors were selected for fragmentation. For the MS2 acquisition, we used a resolution of 15,000 (at *m/z* 200), target AGC value of 1 × 106, maximum injection time of 50 ms, isolation window of 1.2 *m/z*, and fixed first mass at 110 *m/z*. Peptide elution was performed with a nonlinear gradient (flow of 0.300 µL/min during 160 min) using 2% of 80% acetonitrile/0.1% formic acid as solvent B and 98% of 0.1% formic acid as solvent A.

#### 2.3.4. Mass Spectrometry Data Analysis

Proteome Discoverer v2.4 (Thermo Fisher Scientific, San José, CA, USA) was used for peptide and protein identification. For peptide identification, the MS data were searched against the UniProtKB human database (Released 20180207, 42213 sequences, including isoforms). A combination of FF spectral databases built from the MS/MS analysis of the top 14 depleted pools (hSAF spectral library) and the MSPepSearch node plus SEQUEST HT was performed [12]. In addition, we added the human spectral library “ProteomeTools_HCD28_PD” and UniProtKB human database (Date: 28 January 2020), respectively. The search was performed with the following parameters: oxidation of methionine residues and carbamidomethylation of cysteine residues as dynamic and static modifications, respectively. Precursor and fragment ion tolerances were 10 ppm and 0.02 Da. The filters applied FDR < 1% and FDR < 5% confidence at the peptide and protein levels, respectively. The peptide/protein quantification was based on the MS peptide signals (label-free quantification). For label-free quantification, the ‘Minora Feature Detector’ node was included in the processing workflow, and the nodes ‘Precursor Ions Quantifier’ and ‘Feature Mapper’ were included in the consensus workflow.

### 2.4. Bioinformatics and Statistical Analyses

To perform proteomic quantitative analyses, the protein intensities were normalized by log2 transformation and then standardized by subtracting the median of the sample. Statistical analyses were performed using RStudio software [14,15]. Missing values were filtered in order to perform statistical and bioinformatics analyses with proteins quantified in at least 70% of the samples by at least one condition. Dysregulated proteins were determined by applying sparse partial least squares discriminant analysis (sPLS-DA) [16] implemented in the ‘mixOmics’ [17] R package. This is an approach based on a multivariate regression that classifies samples from a high-dimensional dataset while reducing background effects [18]. The expression (log2 intensity) of 850 proteins was used as predictors, with the sampling origin (FF from PCO or non-PCO) as the response. A complementary univariate analysis was performed based on the Student *t*-test (two-tailed), where *p*-values < 0.05 were considered significant. Gene ontology (GO) comparative analysis was performed based on the overrepresentation test, implemented with FunRich v3.1.3 software [19]. Protein classification based on protein class was performed using the PANTHER classification system (www.pantherdb.org, accessed on 22 January 2022) [20]. Pathway enrichment analysis was performed applying the 1D annotation enrichment algorithm proposed by Cox and Mann [21], which was executed with Perseus software [22]. The functional annotation clustering was performed with secreted proteins and well-known folliculogenesis-associated proteins by utilizing the DAVID bioinformatics tool (https://david.ncifcrf.gov/summary.jsp, accessed on 19 January 2022) [23,24]. The same tool was used to know the distribution of the genes coding dysregulated proteins across the distinct chromosomes and find out the significantly enriched chromosomes.

## 3. Results

### 3.1. Protein Identification and Quantification

In this study, a total of 1436 proteins (Table S1) were identified in FF samples (*n* = 20) collected from ovarian SAF of women with PCO (*n* = 5) and women with non-PCO (*n* = 5). An example of the morphological characteristics of the two types of ovaries is shown in Figure 1a. The number of quantified proteins in at least one sample was similar in both groups of samples, which shared 1134 (92%) out of 1239 total quantified proteins (Figure 1b). In general, more than 800 proteins were quantified in each sample, and the coefficient of variation in terms of protein quantification was 4.8% (Supplementary Figure S1). From the 38 proteins uniquely quantified in PCO samples, thioredoxin reductase 1 (TXNRD1) and serine/threonine-protein phosphatase PP1-alpha catalytic subunit (PPP1CA) were quantified in more than 50% of the samples (see Supplementary Table S1). On the other hand, proteins uniquely detected in non-PCO samples were quantified in less than 50% of the samples (except immunoglobulin heavy constant epsilon (IGHE), which was quantified in 50% of the non-PCO FF samples).

Considering that the FF samples were collected from SAF, we compared the list of proteins identified in this study with 24 proteins previously reported by our group as possibly more easily accessed in hSAF [12]. In total, 17 out of 24 proteins were identified in this study. Specifically, VIM, HTRA1, TUBB4B, AMH, TPM4, MDK, WDR1, and PPA1 (eight proteins) were quantified in 100% of the samples. Two proteins, PTMS and HSD17B1, were quantified in at least 50% of the samples of each group, whereafter seven were quantified in less than 50% of the samples in at least one group; these were YBX1, ARF3, LOXL2, S100A6, MYLK, HNRNPH1, and MTPN. The name of the proteins can be found in Table S1.

When analyzing the follicular diameters, no differences were found (*p*-value = 0.87) between the follicles collected from the PCO (mean = 6.8 mm, SD = 1.0) and the non-PCO-groups, respectively (mean = 7.1 mm, SD = 1.1), as shown in Figure 1c.

### 3.2. Dysregulated Protein Profile in FF of hSAF from PCO

**Figure 2:**
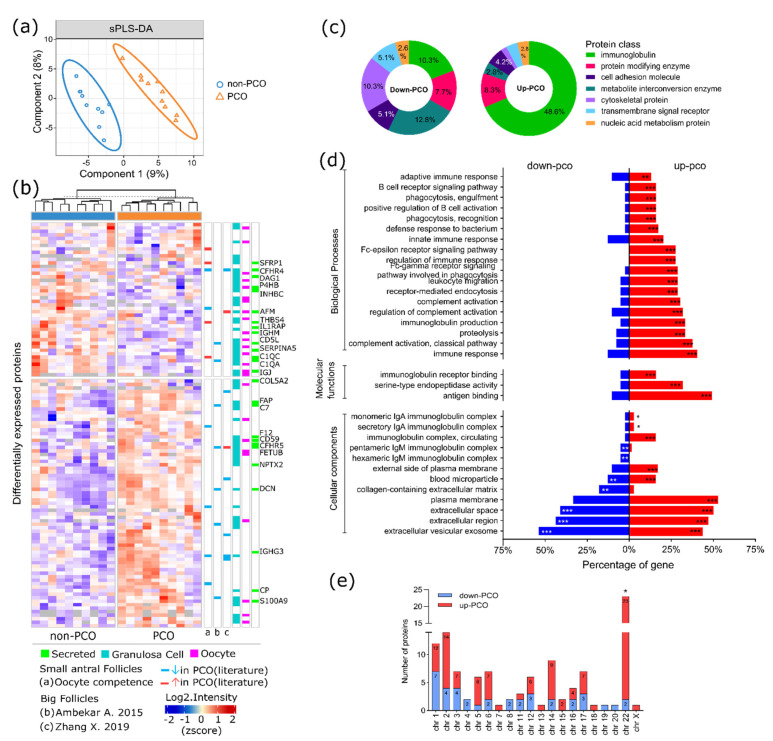
Dysregulated proteins. (**a**) sPLS-DA discriminative analysis carried out with proteins quantified in at least 70% of the samples. (**b**) Unsupervised hierarchical clustering based on the protein expression of 115 dysregulated proteins. Proteins are represented by rows and samples by columns. Blue and red colors in the heat map indicate the respective down- and upregulation of the proteins. Proteins previously reported in two of the largest PCOS proteomic studies [8,9] were highlighted as row annotations. In the same manner, we highlighted granulosa cells (aqua), oocytes (pink), and secreted (green) proteins, as well as proteins involved in oocyte competence from the early stage of follicular development. (**c**) ‘Protein class’ of the dysregulated proteins. (**d**) Gene ontology (GO) enrichment analysis, where significant overrepresented GO terms are indicated with asterisks (* *p* < 0.05, ** *p* < 0.01, and *** *p* < 0.001). (**e**) Chromosomal distribution of the genes coding dysregulated proteins (* *p* < 0.05).

The multivariate discriminative analysis was carried out, with the proteins quantified in at least 70% of the samples showing a significant separation between the PCO and the non-PCO FF samples (Figure 2a). In this case, a total of 115 proteins (Table S2) were differentially expressed, whereafter these were considered as the dysregulated proteins. The discriminative nature of the dysregulated proteins was confirmed upon the unsupervised grouping of the samples according to their biological conditions (Figure 2b, columns). This was achieved by performing an unsupervised hierarchical clustering analysis based on the protein expressions across the samples. The same analysis could also group the dysregulated proteins into two clusters containing the majority of down- and upregulated proteins, respectively (Figure 2b, rows). A complementary univariate analysis was made, based on the Student *t*-test (two-tailed), performed for each protein, where 90% of them presented a *p*-value < 0.05 (*p*-values can be found in Table S2). In total, 40 proteins were found to be downregulated, whereas 75 proteins were upregulated in the PCO FF samples. Interestingly, none of the proteins previously characterized as possibly more concentrated or accessible in FF from hSAF [12] presented differential expression between the PCO and the non-PCO samples.

According to the classification, by ‘protein classes’ (Figure 2c), most of the upregulated proteins were immunoglobulins (48.6%) and protein-modifying enzymes (8.3%). On the other hand, the downregulated proteins were composed of metabolite interconversion enzymes (12.8%), cytoskeletal proteins (10.3%), and protein-modifying enzymes (7.7%). In addition, 10.3% of the downregulated proteins were immunoglobulins. The rest of the downregulated proteins were transmembrane signal receptors (5.1%), cell adhesion molecules (5.1%), and nucleic acid metabolic proteins (2.6%). According to the gene ontology (GO) overrepresentation analysis, most of the dysregulated proteins were extracellular and plasma membrane proteins (Figure 2d, cellular components (CC)). Specifically, the term extracellular vesicular exosome was significantly overrepresented by 54% of the downregulated proteins and 43% of the upregulated proteins (Supplementary Table S3). Particularly, the collagen-containing extracellular matrix was overrepresented by downregulated proteins, while the monomeric and secretory IgA immunoglobulin complexes were enriched by upregulated proteins. In the latter case, these proteins were mostly involved in molecular functions related to antigen binding (49%), serine-type endopeptidase activity (32%), and immunoglobulin receptor binding (16%). The significant biological processes were mainly correlated with upregulated proteins involved in the adaptive and innate immune responses, regulation of complement activation, B-cell receptor signaling, and phagocytosis. The number of dysregulated proteins found per chromosome is shown in Figure 2e. Specifically, chromosome 22 was significantly enriched by upregulated immunoglobulin lambda (variable and constant) proteins and C-Jun-amino-terminal kinase-interacting protein 2 (MAPK8IP2).

### 3.3. Altered Pathways FF of hSAF from PCO

**Figure 3:**
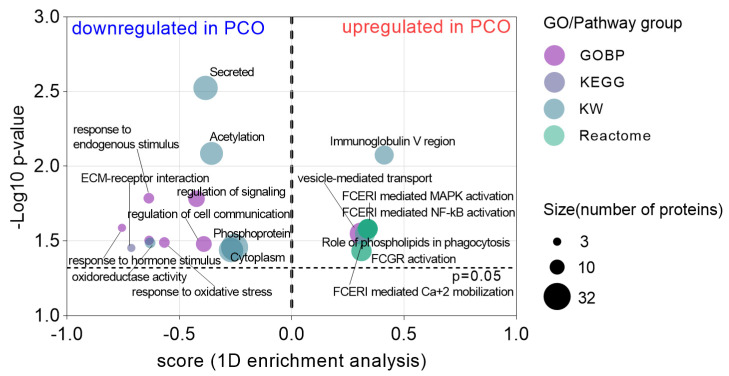
Altered biological pathways in the follicular fluid of polycystic ovaries (PCO). For the analysis we evaluated the KEGG (Kyoto Encyclopedia of Gene and Genome) and Reactome pathways, as well as biological processes (GOBP) and UniProt’s Keywords (KW) (indicated in different colors). The X-axis shows the scores given by the 1D annotation enrichment analysis to each GO/Pathway term. Positive and negative scores indicate down- and upregulation of the GO/pathways and KWs. Y-axis represents the significance grade for each annotation; the larger the -Log10 *p*-value, the more significant the enrichment. Circle size represents the number of protein members found in each GO/pathway/KW.

In an attempt to investigate the biological pathway alterations in FF from PCO’s SAF, a pathway enrichment analysis was performed based on the dysregulated proteins and considering the fold changes between PCO and non-PCO samples. In this analysis, the algorithm tested in every pathway if the corresponding protein’s fold change (FC) inclined to be systematically larger or smaller than the global distribution of the FC for all proteins [21]. The significantly enriched pathways were outlined, and the resulting outcomes are shown in Figure 3. For this particular analysis, we also evaluated the biological processes, as well as the UniProt Keyword (KW) classification. As a result, the extracellular matrix–receptor (ECM–receptor) interaction pathway appeared to be downregulated in the PCO samples. On the other hand, the pathways related to the immune system were found to be upregulated, such as the activation of MAPK and NF-kB that is mediated by the Fc epsilon receptor (FCERI), as well as FCERI-mediated Ca^+2^ mobilization. Among others, the pathways ‘FCGR (Fc gamma receptor) activation’ and ‘Role of phospholipids in phagocytosis’ were also upregulated (see Figure 3). The detailed data on the dysregulated protein members of these pathways can be found in Supplementary Table S4. Interestingly, this analysis showed a downregulation of the biological processes linked to the regulation of signaling and cell communication, as well as the response to hormone stimulus, oxidative stress, and endogenous stimulus (Figure 3 and Table S4).

Another interesting outcome was the enrichment of secreted proteins downregulated in PCO FF samples (Figure 3). Considering the proteins secreted by the GCs and the oocytes, we compared the list of dysregulated proteins with the data obtained by transcriptomics in granulosa cells (GC) and by proteomics in human oocytes [25,26]. From the list of proteins dysregulated in the present study, 25 secreted proteins were previously identified in at least one of these two previous studies. The distribution of these proteins can be observed in Figure 2b (heat map, green color). A total of 16 proteins were downregulated and nine upregulated in the PCO FF samples.

### 3.4. The Functionality of Dysregulated Secreted Proteins

**Figure 4:**
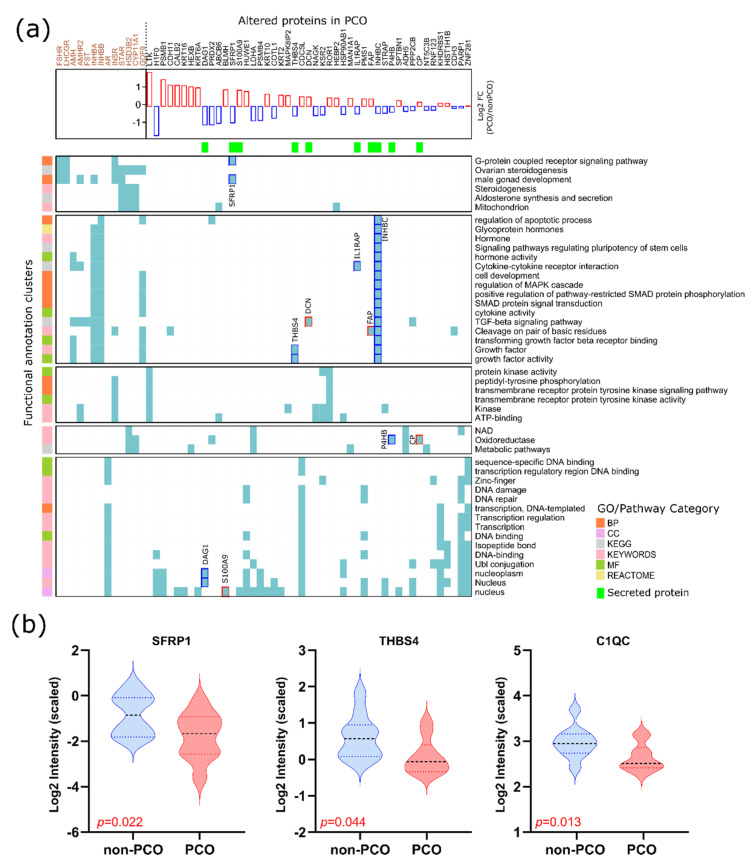
Dysregulated secreted proteins. (**a**) Functional annotation clustering of dysregulated secreted proteins and well-known proteins involved in follicular development (on top, brown color). The analysis was based on the cell component (CC), molecular function (MF), and biological processes (BP) and the KEGG and Reactome pathways, as well as UniProt’s Keyword classification. All of them are indicated in different colors on the left panel. Clustered secreted proteins were highlighted in the central panel in blue (down in PCO) or red (up in PCO). Colored spots in the central panel indicate the corresponding protein (columns)-GO/pathway (rows) association positively reported on public databases. For example, the secreted protein SFRP1, downregulated in PCO, has been positively reported to be associated with the biological processes ‘G-protein coupled receptor signaling pathway’ and ‘male gonad development’. Furthermore, this protein was clustered together with FSHR and LHCGR in a cluster related to steroidogenesis. (**b**) Downregulated secreted proteins in PCO FF that were previously found as downregulated in FF surrounding the oocyte and incapable of maturing to metaphase II in the same type of samples [12].

To investigate the functionality of the secreted proteins, a functional annotation clustering was performed using the DAVID bioinformatics tool (https://david-d.ncifcrf.gov/tools.jsp, accessed on 19 January 2022). In this type of analysis, proteins that share a similar set of GO/pathway terms are most likely involved in similar biological mechanisms [23,24]. To specifically assess the biological functionalities related to folliculogenesis, members of the TGF-β superfamily growth factors such as AMH, AMHR2, GDF9, FST, and FSTL3 were used as input for the analysis. Included were also the gonadotropin receptors FSHR and LHCGR and steroidogenic enzymes CYP19, CYP11A1, HSD3B2, and STAR, as well as the androgen and insulin receptors AR and INSR. All of them were previously evaluated in GC [27] and/or FF [28] of SAF from PCO. As a result, two of the obtained clusters were related to steroidogenesis and the TGF-β-signaling pathway, as well as growth factor/hormone activities (shown in Figure 4, first and second clusters) (Table S5). The downregulated secreted frizzled-related protein 1 (SFRP1) was found to be grouped with the gonadotropin receptors and the steroidogenic enzymes in the cluster associated with steroidogenesis (first cluster). On the other hand, the upregulated secreted proteins decorin (DCN) and prolyl endopeptidase (FAP), as well as the downregulated secreted proteins thrombospondin-4 (THBS4), interleukin-1 receptor accessory protein (IL1RAP), and inhibin beta C chain (INHBC), were grouped within the second cluster. Furthermore, another two clusters came out from the analysis, in which four additional secreted proteins could be included. Protein disulfide-isomerase (P4HB) and ceruloplasmin (CP) were grouped as oxidoreductases in a cluster related to metabolic pathways, whereas dystroglycan 1 (DAG1) and protein S100-A9 (S100A9) were grouped in a cluster related to DNA activity.

From the list of secreted proteins, SFRP1, THBS4 and C1QC (Complement C1q subcomponent subunit C) specifically have previously been identified by our group and associated with oocyte competence already from small antral follicles [12]. In this previous study, the expression of these proteins was decreased in FF surrounding the oocyte incapable of maturing to metaphase II. Interestingly, in the present study, the expression of these proteins decreased significantly in FF from PCO (see Figure 4b).

## 4. Discussion

This work is, to our knowledge, the first high-throughput proteomic study performed on FF from unstimulated hSAF (4.6–9.8 mm) of polycystic ovaries. Compared to other studies, the biological material evaluated in the present study comes from women who did not undergo ovarian stimulation by exogenous gonadotropins, which allowed us to study the FF protein composition in its natural state. This, combined with the small follicular size, constitutes the greatest strength of this study. The study of the molecular dynamics present in SAF of PCO has gained great interest in the last years, since it has been reported that both the oocyte viability and dominant follicle selection are affected already at the early antral stage of follicular development [12,27,28]. We were able to annotate and identify 1436 proteins, which constitute, to our knowledge, a landscape of the largest number of proteins identified in PCO FF samples. Considering that previous PCO proteomic studies have evaluated FF from large follicles, this finding supports the hypothesis that a greater number of proteins can be identified in FF from hSAF compared to larger follicles when using deep-sequencing by high-resolution mass spectrometry platforms [12]. In large follicles, it is more difficult to access the low abundant proteome due to the existence of a large number of highly abundant plasma proteins transferred through the follicular basal membrane in the later phase of folliculogenesis [29,30]. The results obtained from the comparative analysis showed alterations at the protein level in the FF of PCO ovaries related to immune and inflammatory systems, ECM–receptor interaction, collagens-containing the extracellular matrix, regulation of signaling, and response to oxidative stress. Considering the historic link between the Myo-inositol (second messenger of FSH) and PCOS [31], we specifically sought out dysregulated proteins linked to this molecule, and no dysregulated proteins were found in our FF samples. Further analyses such as target proteomics should be done to corroborate the nonexistence of alterations of this molecule in the FF of PCO’s SAF. The use of a discriminative multivariate approach permitted us to consider the relationship between features (proteins), a fact that is ignored when performing univariate analyses such as the *t*-test. Biological systems and signaling pathways are orchestrated by multiple molecules; therefore, large-scale proteomics data are often analyzed by multivariate methods in addition to the univariates [17].

### 4.1. Diminished Cell Signaling and Communication in FF hSAF from PCO

The dysregulation of the plasma membrane, extracellular space, and exosome proteins (Figure 2c) indicate a possible alteration in the regulation of cell–cell communication and, thus, a breakdown in the signaling system designed to ensure crosstalk among paracrine follicular cells. This may explain the downregulation (in PCO FF samples) of biological processes such as the regulation of signaling/cell communication and response to hormone and endogenous stimulus, as well as extracellular matrix (ECM)–receptor interactions (Figure 4). Specifically, the downregulated protein DAG1 was involved in all these processes. This protein acts as a receptor for extracellular matrix proteins containing laminins and is involved in the process of laminin and basement membrane assembly. In a previous proteomic study performed on PCOS FF from large follicles, dysregulation of the basal lamina matrix proteins that included laminins and collagens was reported [32]. Interestingly, the collagen-containing extracellular matrix (ECM) (GO CC) was enriched in our study by downregulated proteins. During normal follicular development, the ECM collagen content decreases as the follicle develops [33,34]. Thus, an alteration in the collagen-containing ECM may contribute to a dysfunction of the follicular growth typically observed in PCO.

### 4.2. Increased Immune System and Inflammatory Processes and Their Link to Oxidative Stress

An increasing number of studies have suggested the role of hormones and immune cells (including both innate and adaptive immune cells) imbalance being part of the PCOS progression [35,36]. In our study, most of the significantly enriched biological processes were immune system processes, including an innate/adaptive immune response, regulation of complements activation, and inflammation. This may constitute additional evidence demonstrating alterations in the FF microenvironment of PCO’s SAF related to an imbalance in the immune system and its consequences. Curiously, exosome proteins, which were dysregulated in our study, have been identified to function, among other processes, in inflammatory pathways and reproduction [37]. Specifically, protein S100A9 (upregulated in our PCO samples) was previously found to be upregulated in exosomes derived from PCOS’s FF and linked to inflammatory processes and the disruption of steroidogenesis via activation of the nuclear factor-kappa B (NF-κB) signaling pathway (proinflammatory pathway) [7]. In accordance with this finding detected in large follicles, an upregulation of the Fc epsilon receptor (FCERI)-mediated NF-κB activation pathway, which is critical for proinflammatory cytokine production, was observed in our samples (FF from SAF) (see Figure 3 and Table S4). Interestingly, González et al. [38] found that increased activation of NF-κB factor in mononuclear cells triggers inflammatory signals that induce insulin resistance and hyperandrogenism in PCOS (independently of obesity). The same research group reported a link between the generation of reactive oxygen species (ROS)-induced oxidative stress and the development of insulin resistance and hyperandrogenism in PCOS [39]. Although these associations were found in mononuclear cells, a recent model proposed by Liu et al. [36] in large follicles suggested that inflammatory cytokines derived from the peripheral circulation enter into the follicles through the ovarian circulation system and activate the NF-κB factor in FF, which, in turn, seems to promote the activation of inflammatory pathways in GCs. In addition, mitochondrial damage is provoked by the GC inflammatory cascade, which exacerbates the generation of ROS and, thereby, leads to a reduction of cell proliferation, ultimately affecting the growth and development of oocytes. The results from our study suggest that similar dysfunctions may occur already from SAF in PCO, since biological processes, such as a response to oxidative stress and oxidoreductase activity (involved proteins: PRDX2, PPP2CB, P4HB, ATRN, and PARP1), were found to be downregulated in PCO’s FF samples (Figure 3). In addition to the biological processes found as enriched, a significant overrepresentation of upregulated immunoglobulin lambda proteins was observed in chromosome 22. As far as we know, no previous studies have reported anomalies in chromosome 22 associated with PCOS. However, it is well-known that immunoglobulin lambda proteins participate in several key processes of the immune system and stimulate local inflammatory responses [40].

### 4.3. Altered Secreted Proteins and Their Role in Oocyte Competence

Considering that identified secreted proteins could originate from the oocyte and somatic follicular cells intended to ensure a continued communication flow during follicular development, we investigated their functionality through a bioinformatics functional annotation clustering. Interestingly, the SFRP1 protein was included in a cluster associated with steroidogenesis. The SFRP family of proteins functions as modulators of Wnt signaling. Recently, a study performed in GCs from rats demonstrated that one of the SFRP family proteins (SFRP-4) functions via the modulation of β-catenin and PKB/AKT activation in [41] steroidogenesis [41]. Specifically, SFRP1 is known to bind and inhibit the activity of RANKL (receptor activator of nuclear factor kappa-Β ligand) [42], a member of the TNF family, which was found to inhibit steroidogenesis in undifferentiated ovarian cells [43]. On the other hand, the expression of SFRP1 was previously found to increase in the FF microenvironment of oocytes capable of maturing to metaphase II (i.e., downregulated in FF surrounding the oocyte incapable of maturing to metaphase II) [12]. However, in the present study, SFRP1 appeared downregulated in PCO’s FF samples (Figure 4b). The same occurred with secreted proteins THBS4 and C1QC. This may indicate that the downregulation of these proteins in the FF of hSAF may affect future oocyte competence.

Within the cluster associated with TGF-β signaling and growth factor/hormone activities, DCN, a secreted proteoglycan, may exert paracrine actions via growth factor receptor systems [44]. On the other hand, the downregulation of THBS4 seems to affect growth factor activities, as shown in Figure 4a. The decreased expression of INHBC in PCO may indicate a dysfunction in gonadal hormone secretion, as well as cell growth and differentiation.

In general, two limitations were considered in our study. Considering the relatively low number of patients included in this study, our research was contemplated as a pilot study that served as a proof of concept to demonstrate that proteomic changes related to PCO can be found and occur in FF of SAF. However, further experiments should be done with a larger number of patients to confirm our current findings. Additionally, women with PCO included in this study did not get a complete diagnosis of PCOS. At the time of ovarian tissue cryopreservation, a PCOS diagnosis was not required, since the priority was fertility preservation. However, the morphological characteristics of the polycystic ovaries included in this study (Figure 1a), together with hormone profiles (Table 1) of the patients, are distinctive of PCOS.

In conclusion, our study revealed, for the first time, evidence of proteomic alterations in PCO FF of hSAF that may be related to PCOS dysfunctions related to follicular growth and subsequent oocyte competence. The proteome profile of the PCO FF samples indicated an aberrant increase of the immune and inflammatory processes linked to a possible failure of the response to oxidative stress. In addition, diminished cell signaling and communication in the PCO FF samples may indicate alterations at the early follicular stage related to follicular growth and development. The dysregulation of some secreted proteins may be related to a subsequent impaired oocyte competence as a consequence of malfunction in steroidogenesis, cytokine activity, and DNA repair.

## Figures and Tables

**Table 1:** Patient demographics and clinical information.

	Non-PCO	PCO
Women (N)	5	5
Age in years (mean ± SD)	27.4 ± 5.5	25.1 ± 4.4
Ovarian volume in mL (mean ± SD)	8.5 ± 1.1	16.5 ± 3.5
Median number of FF/woman (range)	5.6 (5–6)	9.8 (7–14)
Follicular diameter in mm (mean ± SD)	7.1 ± 1.1	6.8 ± 1.0
AMH in pmol/L (mean ± SEM)	16.8 ± 3.5	39.1 ± 16.8
FSH in IU/L (mean ± SEM)	5.0 ± 1.9	5.6 ± 1.6
LH in IU/L (mean ± SEM)	5.0 ± 2.5	10.3 ± 6.4
LH/FSH ratio (mean ± SEM)	1.0 ± 0.3	1.7 ± 0.9

## Data Availability

The mass spectrometry proteomics data have been deposited to the ProteomeXchange Consortium via the PRIDE [45] partner repository with the dataset identifier PXD031485.

## Supplementary Materials

The following supporting information can be downloaded at https://www.mdpi.com/article/10.3390/life12030391/s1: Figure S1: Number of proteins quantified per sample. Table S1: List of identified proteins. Table S2: Dysregulated proteins. Table S3: GO overrepresentation analysis performed with dysregulated proteins. Table S4: Biological pathways and processes enriched by dysregulated proteins. 1D annotation enrichment analysis. Table S5: Functional annotation clustering of dysregulated secreted proteins.
