# Supplementary figures and images for: Proteomic Alterations in Follicular Fluid of Human Small Antral Follicles Collected from Polycystic Ovaries—A Pilot Study
Source: Life (Basel). 2022 Mar 8;12(3):391. doi: 10.3390/life12030391 (PMC8954146; doi:10.3390/life12030391)

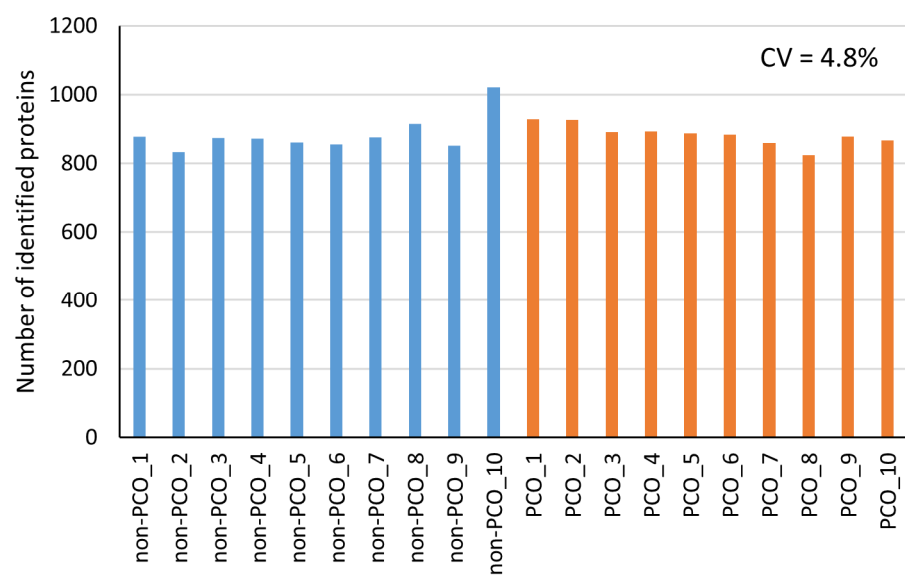

Figure S1: Number of proteins quantified per sample.

Supplement: Supplementary file 1 [file life-12-00391-s001.zip › supplementary figure S1.pdf]
